# Retinal Oximetry with Scanning Laser Ophthalmoscope in Infants

**DOI:** 10.1371/journal.pone.0148077

**Published:** 2016-02-03

**Authors:** Wouter B. Vehmeijer, Vigdis Magnusdottir, Thorunn S. Eliasdottir, Sveinn Hakon Hardarson, Nicoline E. Schalij-Delfos, Einar Stefánsson

**Affiliations:** 1 Department of Ophthalmology, University of Leiden, Leiden University Medical Center, Leiden, Netherlands; 2 Department of Ophthalmology, Oslo University Hospital, Oslo, Norway; 3 Department of Ophthalmology, University of Iceland, Landspítali University Hospital, Reykjavik, Iceland; University of Florida, UNITED STATES

## Abstract

**Purpose:**

Dual wavelength retinal oximetry has been developed for adults, but is not available for infants. Retinal oximetry may provide insight into the pathophysiology of oxygen-mediated diseases like retinopathy of prematurity. More insight in the oxygen metabolism of the retina in infants may provide valuable clues for better understanding and subsequent prevention or treatment of the disease. The measurements of oxygen saturation are obtained with two fundus images simultaneously captured in two different wavelengths of light. The comparison in light absorption of oxygenated and deoxygenated hemoglobin can be used to estimate the oxygen saturation within the retinal vessels by means of a software algorithm. This study aims to make retinal oximetry available for neonates. The first step towards estimating retinal oxygen saturation is determining the optical density ratio. Therefore, the purpose of this study is to image healthy newborn infants with a scanning laser ophthalmoscope and determine the optical density ratio for retinal oximetry analysis.

**Methods:**

Images of the retina of full-term healthy infants were obtained with an SLO, Optomap 200Tx (Optos), with two laser wavelengths (532nm and 633nm). The infant lay face down on the lower arm of the parent, while the parent supported the chest and chin with one hand, and stabilized the back with the other hand. No mydriatics or eyelid specula were used during this study. The images were analyzed with modified Oxymap Analyzer software for calculation of the Optical Density Ratio (ODR) and vessel width. The ODR is inversely and approximately linearly related to the oxygen saturation. Measurements were included from the superotemporal vessel pair. A paired *t-*test was used for statistical analysis.

**Results:**

Fifty-nine infants, (58% female), were included with mean gestational age of 40 ± 1.3 weeks (mean ± SD) and mean post-natal age of 16 ± 4.8 days. A total of 28 images were selected for retinal oximetry analysis. The ODR was 0.256 ± 0.041 for the arterioles and 0.421 ± 0.089 for the venules (*n* = 28, *p* < 0.001). The measured vessel-width for the arterioles was 14.1 ± 2.7 pixels and for the venules 19.7 ± 3.7 pixels (*n* = 28, *p* < 0.001).

**Conclusions:**

Retinal oximetry can be performed in newborn infants by combining an SLO and a dual-wavelength algorithm software. Sensitivity of the approach is indicated by the fact that the ODR measurements are significantly different between the arterioles and the venules. However, more variability in ODR is seen with the SLO approach in babies than is seen with conventional oximetry in adults. This approach is completely non-invasive, non-contact and even avoids the use of mydriatics or eyelid specula.

## Introduction

The etiology of retinopathy of prematurity (ROP) is multifactorial. Among the most important risk factors are premature birth, low birth weight, duration of artificial ventilation and supplemental oxygen.[1–3] Oxygen plays a major role in the pathophysiology of ROP. Patz et al.,[4] concluded that administering high doses of oxygen to prematurely born infants could lead to ROP. Since then, several large clinical trials (BOOST, SUPPORT, BOOST II, COT) were performed to investigate the optimal amount of oxygen supplementation for neonatal care and prevention of ROP.[5–8] To measure the relative oxygen saturation a pulse oximeter is used, providing information about oxygen saturation levels in the peripheral but not in the central circulation.

In the pathophysiology of ROP, two phases have been recognized. The first, hyperoxic, phase occurs shortly after birth due to an abundance of oxygen, as compared to the intrauterine situation, which causes suppression of oxygen-regulated angiogenic growth factors resulting in cessation of retinal vessel growth. As the retina develops, the oxygen need of the retina transcends the oxygen supply by the retinal vessels and the second, hypoxic, phase occurs. This initiates a cascade in which retinal vessel growth is stimulated. Excessive stimulation results in pathologic dilation of central retinal vessels and neovascularization as seen in more severe ROP, leading to retinal detachment in the worst cases.[9] The disease can be treated, when detected in time, but this is not always successful.[10] It is clear that oxygen plays an important role in the development of ROP, but much is yet unknown. More insight in the oxygen metabolism of the retina in infants may provide valuable clues for better understanding and subsequent prevention or treatment of the disease. Unfortunately, no method is currently available to measure the oxygen saturation within the retinal vessels in infants or premature born babies.

Dual wavelength retinal oximetry allows for non-invasive measurement of the oxygen saturation as well as vessel diameter of the retinal vessels. Currently, there are two commercially available oximeters (Oxymap ehf., Reykjavik, Iceland and Imedos, Jena, Germany). Both these oximeters utilize the optics of conventional fundus cameras. The measurements of oxygen saturation are obtained with two fundus images simultaneously captured in two different wavelengths of light. The comparison in light absorption of oxygenated and deoxygenated hemoglobin can be used to estimate the oxygen saturation within the retinal vessels by means of a software algorithm.[11]

Imaging the retina of infants is currently used to objectively screen and monitor retinal vessel development. The most commonly used device for this purpose is the RetCam (Clarity Medical Systems, Pleasanton, CA, USA). The Scanning Laser Ophthalmoscope (SLO), Optomap 200Tx, (Optos plc., Dunfermline, Scotland, UK) has been used as a non-contact, widefield camera in a case series to image the retina of infants.[12] With minimal modification to the system, the Optos device can function as a retinal oximeter, as it also provides images with different wavelengths, similar to the Oxymap T1 system.[12, 13] Several studies[14–17] have been conducted to demonstrate the use of an SLO to measure oxygen within the blood vessels in animals and adult humans. Recently a study by Kristjansdottir et al.[13] with a combination of the Optomap 200Tx, SLO and the dual-wavelength oximetry software (Oxymap ehf., Reykjavik, Iceland) was used to estimate retinal oxygen saturation within the retinal vessels in adults. These measurements of oxygen saturation were both sensitive and repeatable as shown in studies of adult patients with central retinal vein occlusion (CRVO) and healthy volunteers breathing different concentrations of oxygen.[18] Retinal oximetry has not previously been performed in children. No data are therefore available on the saturation within the retinal vessels in children. However, gaining insight in saturation levels within the retinal vessels may be valuable in vascular retinal diseases in children like Coats disease, Familial Exudative Vitreo-Retinopathy and especially in premature neonates with the risk of developing ROP.[19–21] This study aims to make retinal oximetry available for neonates. The first step to estimate the retinal oxygen saturation is determining the optical density ratio of the retinal vessels. Therefore, the purpose of this study is to image healthy newborn infants with a scanning laser ophthalmoscope and determine the optical density ratio for retinal oximetry analysis.

## Methods

The study was approved by the National Bioethics Committee of Iceland and the Icelandic Data Protection Authority, and adhered to the tenets of the Declaration of Helsinki. The parent or legal guardian of the infants provided written informed consent before participation in the study.

### Study Population

Infants were recruited at the pediatric department of the Landspítali, University Hospital, Reykjavik, Iceland, during the period of the first of September until December 2014. Infants were recruited during the “five days post-partum” examination. This national examination program by the pediatric department of the Landspítali screens newborn children in Iceland during the first week post-partum. For inclusion, the infants needed to be healthy, as well as have a gestational age at birth between 37–42 weeks with an age appropriate weight for gestational age and no complications during birth.

### Study Protocol

**Fig 1:**
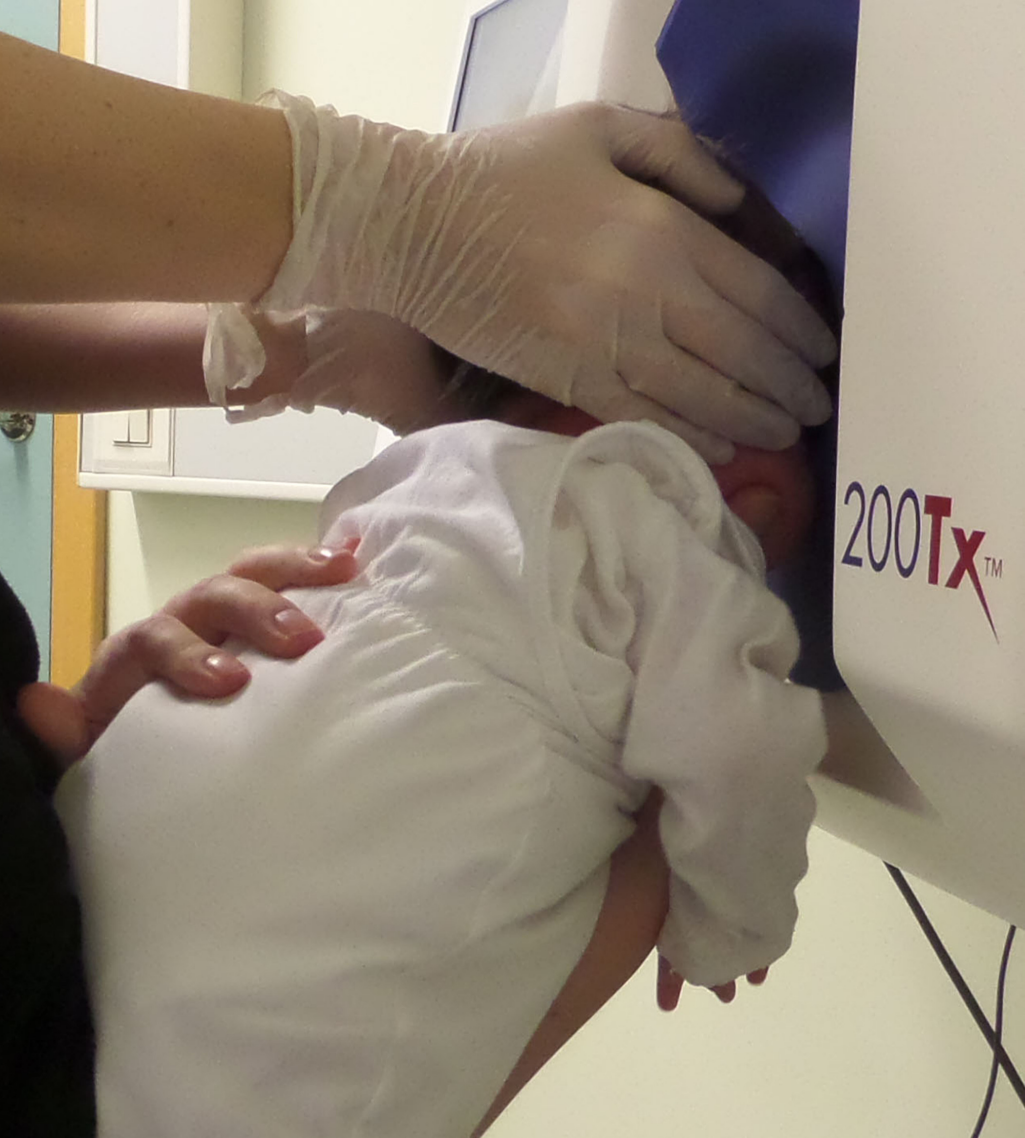
Modified-flying baby position for alignment with the Scanning Laser Ophthalmoscope.

After written informed consent was acquired, the parent was seated before the Optomap 200Tx (Optos plc., Dunfermline, Scotland, UK) and instructed how to hold the infant in a modified flying baby position, previously described.[12] The infant lay face down on the lower arm of the parent, while the parent supported the chest and chin with one hand, and stabilized the back with the other hand (Fig 1). No mydriatics or eyelid specula were used during this study. Assisting the parent, the researcher aligned the head of the infant towards the SLO with the aid of the visual feedback of the monitor of the Optomap. During the alignment of the head, the eyelids were spread by hand either with a cotton tip or rubber glove. If alignment was optimal the photographer captured the image. For each infant, one of the eyes was imaged. Fifty-nine infants, (58% female), were included at a mean gestational age of 40 weeks and mean post-natal age of 16 days (Table 1). Images were acquired from 55 infants, (3 infants did not cooperate, 1 infant was excluded due to premature birth). During a single imaging session, both Ultra-Widefield (200°) and ResMax (100°) images were acquired. For this study, the images in the ResMax setting were used for retinal oximetry analysis. A total of 250 ResMax (100°) retinal images were captured with a median of 4 images per infant (range 0–8).

**Table 1:** Baseline Characteristics of the Subjects.

Characteristics	Subjects
	*n = 59*
Gender male/female	25 (42%) / 34(58%)
Gestational Age *(weeks)*	40 ± 1.3*
Post natal age *(days)*	16 ± 4.8*
Birth weight *(g)*	3729 ± 456*
Length *(cm)*	51.4 ± 1.9*

* (mean±SD).

The Optomap 200Tx uses two wavelengths of light (532 nm (green) and 633 nm (red)) to capture two images of the retina and its vessels. The light absorbance of oxy- and deoxyhemoglobin is similar at 532nm, which was therefore used as a reference wavelength. At the sensitive wavelength of 633nm, the light absorbance of deoxygenated hemoglobin is about 8 times higher than absorbance of oxygenated hemoglobin.[22] These characteristics are required for the use of dual-wavelength oximetry. A dual-wavelength software algorithm processes both images to calculate the Optical Density (OD) (Eq 1). The ratio between the OD of the sensitive wavelength and the isosbestic wavelength is called the optical density ratio, ODR (Eq 2). The ODR is inversely and approximately linearly related to the oxygen saturation.[11, 23, 24]

**Fig 2:**
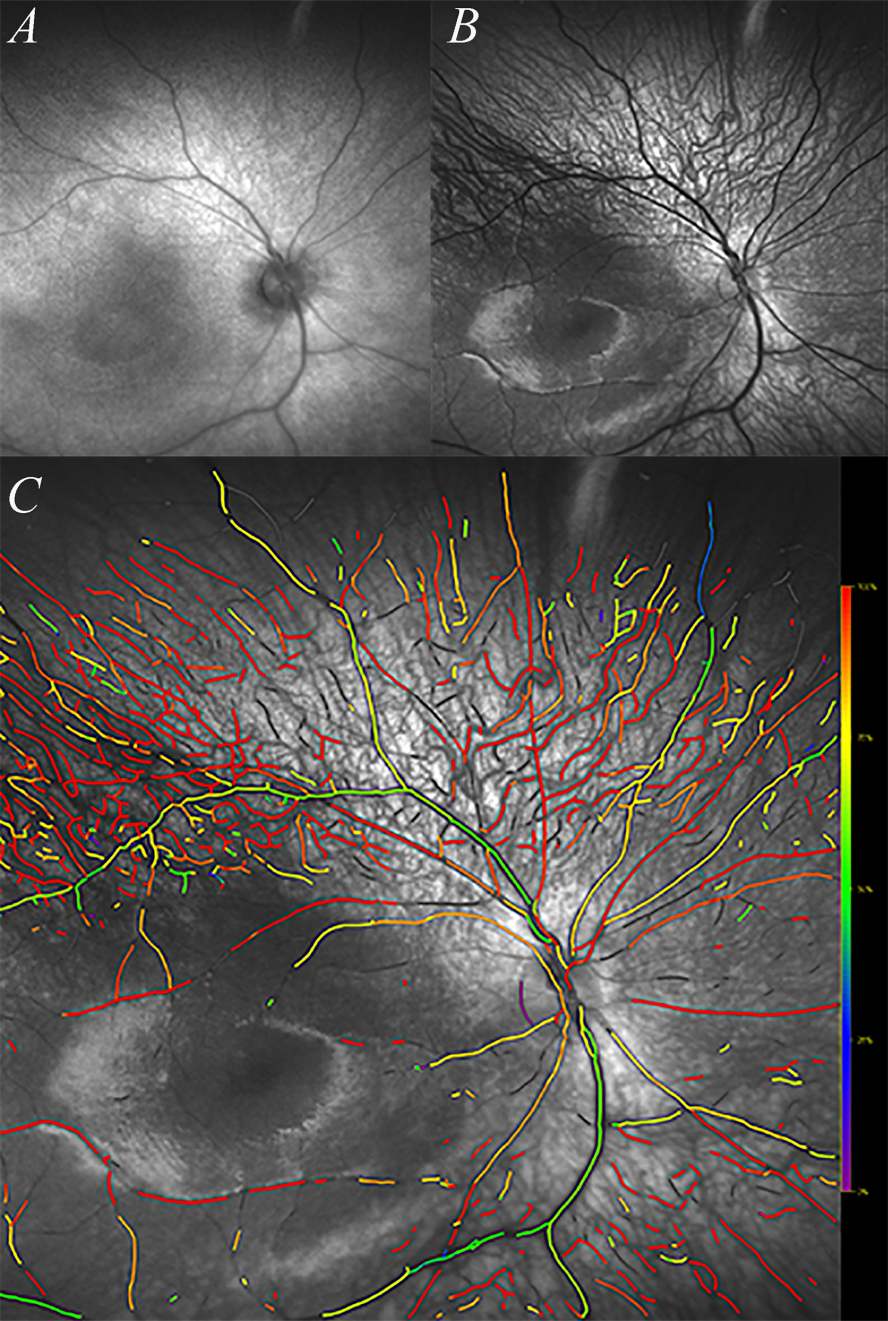
Images of the fundus obtained with two laser wavelengths (A, 633nm; B, 532nm) Third image (C) is pseudocolor fundus image created by Oxymap algorithm. The colors denote approximate oxygen saturation. However, the calibration is set for an adult population and it is likely that the colors do not represent true oxygen saturation in infants. In addition to the retinal vessels, the vessels of the choroid can be seen in the superotemporal, superonasal and inferior-nasal quadrant

The ResMax images were processed by a modified version of the dual-wavelength oximetry algorithm, (Oxymap Analyzer Software, Oxymap ehf., Reykjavik, Iceland) and calculations are plotted in a pseudocolor fundus images (Fig 2). The modification of the software was for the spectral imaging and wider degree (100°) images. Secondly, the Ultra-Widefield images (200°) were obtained for further research purpose.

$$\mathrm{OD}=\log(\frac{I_{0}}{I})$$ (1)

$$\mathrm{ODR}=\;\frac{{\mathrm{OD}}_{633}}{{\mathrm{OD}}_{532}}$$ (2)

For analysis, images of each infant were selected if the optic disc and the superotemporal vessel pair (arterioles and venules) were in frame. In case the images were out of focus, they were disregarded for analysis. Additionally, if multiple images for a single subject were within these criteria, the image with the best contrast and focus was selected. Due to these selection criteria, 28 images were selected for retinal oximetry analysis from the total of 250 ResMax images acquired during the imaging sessions. The image analysis was standardized for this study. The ODR was measured in the main superotemporal vessel pair from the rim of the optic disc until the first branching of the vessel. Selecting the main superotemporal vessel pair was done manually and if vessel segments were intertwined, these vessel segments were excluded from analysis. The vessel width had to be larger than 6 pixels and a vessel length had to be between 60 pixels and 350 pixels to be included. The first 15 pixels from the rim of the optic disk were excluded from measurement. Second, if the vessel segment between the rim of the optic disk and the first branching point was shorter than 60 pixels, it was excluded and the vessel segment from the first branching point until the second branching point was measured, provided that the second segment was 60 pixels or more. The outcomes are reported as mean ± SD.

### Statistical Analysis

Statistical analysis was performed using SPSS version 22 (Release 22.0.0.0, IBM). A paired *t*-test was applied to determine if the difference in oxygen saturation in the retinal superotemporal vessel pair is statistically different.

## Results

**Fig 3:**
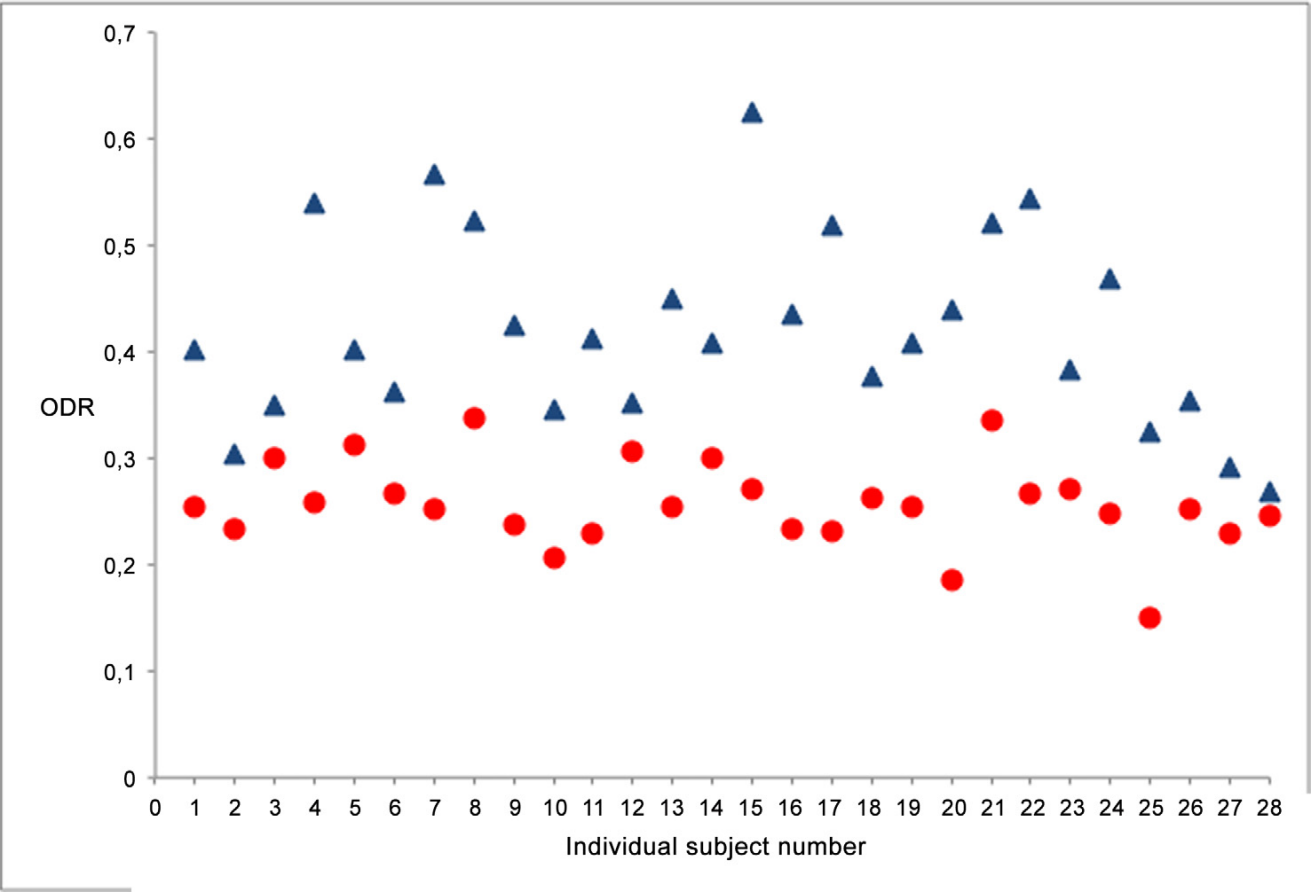
The scatterplot displays the ODR (optical density ratio) of the main retinal superotemporal vessel pair. Arterioles (red circle) and venules (blue triangle) are significantly different (p < 0.001, paired *t* -test).

The ODRs were calculated for the superior vessel pair of a total of 28 ResMax images. These results are displayed in Fig 3. The mean ODR was 0.256 ± 0.041 with a median of 0.255, range 0.150–0.337 for the arterioles and for the venules 0.421 ± 0.089, with a median of 0.409, range 0.268–0.626. The difference between the ODR of the arterioles and the venules is statistically significant according to a paired *t*-test (*n =* 28, *p* < 0.001). The measured vessel-width for the arterioles was 14.1 ± 2.7 pixels and for the venules 19.7 ± 3.7 pixels (*n* = 28, *p* < 0.001). Table 2 provides an overview of the results.

**Table 2:** Optical Density Ratio (ODR) and Vessel Width of the Retinal Vessels.

**ODR** *(n =* 28)	Mean ± SD	Median	Range	*p—*value
Arterioles	0.256 ± 0.041	0.255	0.150–0.337	
Venules	0.421 ± 0.089	0.409	0.268–0.626	*p <* 0.001
**Diameter** *(Pixels****)***				
Arterioles	14.1 ± 2.7	13.5	9.9–20.3	
Venules	19.7 ± 3.7	20.1	14.0–25.5	*p* < 0.001

## Discussion

Retinal oximetry can be performed in newborn infants by combining the Optomap 200Tx SLO and a modified version of the Oxymap dual-wavelength algorithm software. This is the first proof of principle of SLO oximetry in an infant population. Sensitivity of the approach is indicated by the fact that the ODR (Optical Density Ratio) measurements are significantly different between the arterioles and the venules. This approach is completely non-invasive, non-contact and even avoids the use of mydriatics or eyelid specula.

Thus far, clinical retinal oximetry studies have mostly been conducted in adults with conventional fundus cameras. Kristjansdottir et al. [13] conducted a pilot study on retinal oximetry with the Optomap 200Tx in adults. In children, retinal oximetry studies have not been performed. The oxygen saturation within the retinal vessels, can be calculated by the formula *SatO*_2_ = (*a* ∙ *ODR* + *b*) ∙ 100%. This equation allows for calculation of oxygen saturation percentage from ODR with two constants (*a* and *b)*. Both constants have been determined for adults, by matching the arteriolar and venular ODRs measured with the SLO in adult healthy subjects to saturation values found in a previous study by Schweitzer et al.[25] with a calibrated device.[13] Currently, the results of the retinal oximetry studies are all from an adult population and therefore the calibration factors are not applicable for the use in infants. More research needs to be conducted to determine the exact calibration factors in an infant population, to transform the ODR to reliable saturation values. The results are therefore, presented as ODR here. The ODR in infants is higher for both arterioles (0.256) and venules (0.421) in comparison to the mean adult ODR values from the same instruments 0.210 for arterioles and 0.351 for venules. The measured ODR values (and calculated saturation) were quite variable between individuals as can be seen from standard deviations and from Fig 3. Variability could potentially be decreased with optimization of the imaging lasers. The current laser setup of the Optomap is optimized for colour images. The ODR is more variable in venules in comparison to arterioles. This phenomenon has been reported in previous papers.[26–28] A part of the reason may be physiological, i.e. more factors affect the saturation in the outflowing blood in the venules then the inflowing blood. Technical reasons may also lead to more variability in measurements of the (dark) venules, compared to the (bright) arterioles but this has to be studied further.

The eye of a newborn child is different from the adult. At birth, hemoglobin consists for 60–80% of fetal hemoglobin.[29] Fetal hemoglobin has slightly higher absorption characteristics for oxygen and light.[30] However, this is unlikely to have a large effect on retinal oximetry because the light absorbance of the wavelengths used is very similar for adult and fetal hemoglobin. Secondly, there are differences in the dimensions of the eye, such as the axial length. The axial length of a newborn full term infant’s eye is on average 17.02 mm, increasing 5.05 mm over three years with an orbital volume increase form 7cc of a newborn to 30cc in adulthood.[31, 32] In addition, retinal pigmentation increases during infancy.

The study by Patel et al., demonstrated the use of SLO in a case series of infants with ROP.[12] This study and the current study found that a good overview of the retina of infants could be achieved. Patel et al., concluded that the quality imaging with the Optomap was superior to the Retcam. As an advantage, the SLO has the ability to image the retina at either 100 degrees (ResMax) or 200 degrees (Ultra-Widefield) with current neonatal cameras a maximum angle of 130 degrees can be achieved. Secondly, with the SLO approach, the light exposure only occurs during the actual scanning of the retina, whereas the current neonatal cameras use continuous illumination. In addition the SLO has the possibility to capture retina images without making contact to the eye or the use of mydriatics. However, images can not be acquired in supine position, only by holding the infant in front of the scanning head of the SLO, this could be undesirable in clinical situations with severely ill neonates. In contrast to the study of Patel, the current study was performed without the use of mydriatics or an eyelid speculum, minimizing the distress for the infants. The lack of mydriatics or eyelid speculum may have had an effect on the image quality. Therefore, it is likely that the use of an eyelid speculum and mydriatics in neonates will improve the image quality (contrast) as well as the reliability of the measurements.

In summary, this novel approach of the use of an SLO and the dual-wave length software algorithm is applicable to perform non-invasive, retinal oximetry in infants. The use of SLO has several advantages in comparison to conventional fundus imaging. However, the SLO approach is associated with high variability. In the future, technical improvement to the hardware, such as changing the laser setup of the Optomap could improve the precision of the Optomap as an oximeter. Software modifications may also reduce this variability. SLO retinal oximetry may in the future provide a means to monitor the saturation directly in the retinal vessels of (premature) infants allowing for better insight in the oxygen distribution in the retina. The increase in insight of the oxygen distribution in the retina can lead to a better understanding of the pathophysiological cascade of ROP and to an improvement of management and treatment modalities of ROP.

## Supporting Information

S1 File Specific data for all individuals (XLSX)
